# Mapping behaviorally relevant light pollution levels to improve urban habitat planning

**DOI:** 10.1038/s41598-019-48118-z

**Published:** 2019-08-15

**Authors:** Aaron E. Schirmer, Caleb Gallemore, Ting Liu, Seth Magle, Elisabeth DiNello, Humerah Ahmed, Thomas Gilday

**Affiliations:** 1 Northeastern Illinois University, Dept. of Biology, 5500 St. Louis Ave., Chicago, IL 60625 USA; 2 Lafayette College, International Affairs Program, 730 High St., Easton, PA 18042 USA; 3 Northeastern Illinois University, Dept. of Geography and Environmental Studies, 5500 St. Louis Ave., Chicago, IL 60625 USA; 4 Lincoln Park Zoo, Urban Wildlife Institute, 2001 N Clark St, Chicago, IL 60614 USA

**Keywords:** Urban ecology, Sustainability, Environmental impact

## Abstract

Artificial nighttime lights have important behavioral and ecological effects on wildlife. Combining laboratory and field techniques, we identified behaviorally relevant levels of nighttime light and mapped the extent of these light levels across the city of Chicago. We began by applying a Gaussian finite mixture model to 998 sampled illumination levels around Chicago to identify clusters of light levels. A simplified sample of these levels was replicated in the laboratory to identify light levels at which C57BL/6J mice exhibited altered circadian activity patterns. We then used camera trap and high-altitude photographic data to compare our field and laboratory observations, finding activity pattern changes in the field consistent with laboratory observations. Using these results, we mapped areas across Chicago exposed to estimated illumination levels above the value associated with statistically significant behavioral changes. Based on this measure, we found that as much as 36% of the greenspace in the city is in areas illuminated at levels greater than or equal to those at which we observe behavioral differences in the field and in the laboratory. Our findings provide evidence that artificial lighting patterns may influence wildlife behavior at a broad scale throughout urban areas, and should be considered in urban habitat planning.

## Introduction

Population growth, economic development, and urbanization have increased the density and extent of artificial lights in natural, seminatural, and urban settings^1–3^, leading to concerns about “ecological light pollution^4^.” Though ecologists, astronomers, and other interested parties demonstrate that increases in artificial nighttime light represent a significant component of global change^5,6^, a general lack of public concern^7,8^ has resulted in limited research to inform antiphotopollution policies^9^. Similarly, while urban wildlife has garnered increased attention^10^, studies investigating the effects of altered lighting on wildlife in urban landscapes, particularly on terrestrial mammals, are relatively scarce. Improved understanding of how urban wildlife reacts to modified light levels can support management and conservation. Urban wildlife faces numerous novel stressors, ranging from roads and traffic, to new competitors and predators, to direct interactions with humans^11^, as well as altered lightscapes. Despite excess light becoming an important component of global environmental change^1,2,9^, we are just beginning to understand photopollution’s effects^9^. Light is a powerful and consistent environmental signal that communicates important daily and seasonal changes in an organism’s environment^12,13^. It is associated with numerous biological and ecological impacts, potentially acting as a novel evolutionary selection pressure^14^. For wildlife, light influences navigation, activity, and reproduction. Many studies document artificial lighting’s contribution to hatchling turtle and avian mortality due to disorientation^15–20^. Other well documented effects include temporal niche partitioning; altered repair and recovery of physiological function; interference with detection of predators and environmental resources, signaling, and camouflage; changes in reproductive behavior; and alterations in circadian rhythms^3,4,14^. In laboratory studies, slight changes to nocturnal light levels, in some cases just above natural moonlight^21,22^, alter hormone (melatonin) secretions, endogenous circadian rhythm waveforms, levels of daily locomotor activity, reproductive and immune function, and body mass^21–23^.

Remote sensing is an efficient way to model outdoor artificial lighting over large metropolitan areas^24–27^, but global satellite products’ coarse spatial resolutions limit their capability to identify small-scale variations of light intensity within urban areas^28,29^. Various dedicated airborne sensors (e.g., Airborne Visible/Infrared Imaging Spectrometer (AVIRIS)) provide high spatial and spectral resolution nighttime image data^30,31^. Few of these, however, are publicly available. A notable exception is NASA’s published images of the Earth taken by astronauts from the International Space Station (ISS). Due to changes in orbit and angle, the spatial resolution of these images can range from 1 km, more typical of “lights at night” data available from satellite imagery, down to 1 m, giving some photographs resolutions more suitable for city-scale studies than existing publicly available satellite data^28^.

While there has been considerable work on artificial nighttime lighting using satellite data to assess habitat erosion at the landscape scale^2,32,33^, few studies use remotely sensed data to directly study artificial light’s impacts on wildlife behaviors^34,35^. To our knowledge, there are no studies that use such techniques to connect behaviorally relevant artificial light levels observed in laboratory settings to observational data. Here, we present a highly cost-efficient approach (see Fig. S1 for a graphical overview of the process) that combines laboratory and observational data to derive maps of behaviorally relevant illumination for a specific subset of large mammalian taxa common to Chicago, Illinois, USA. We use estimates of these levels to assess the degree to which artificial light degrades greenspace within the city.

## Results

### Aerial photography reveals that ground-level lighting varies across the Chicagoland region

**Figure 1:**
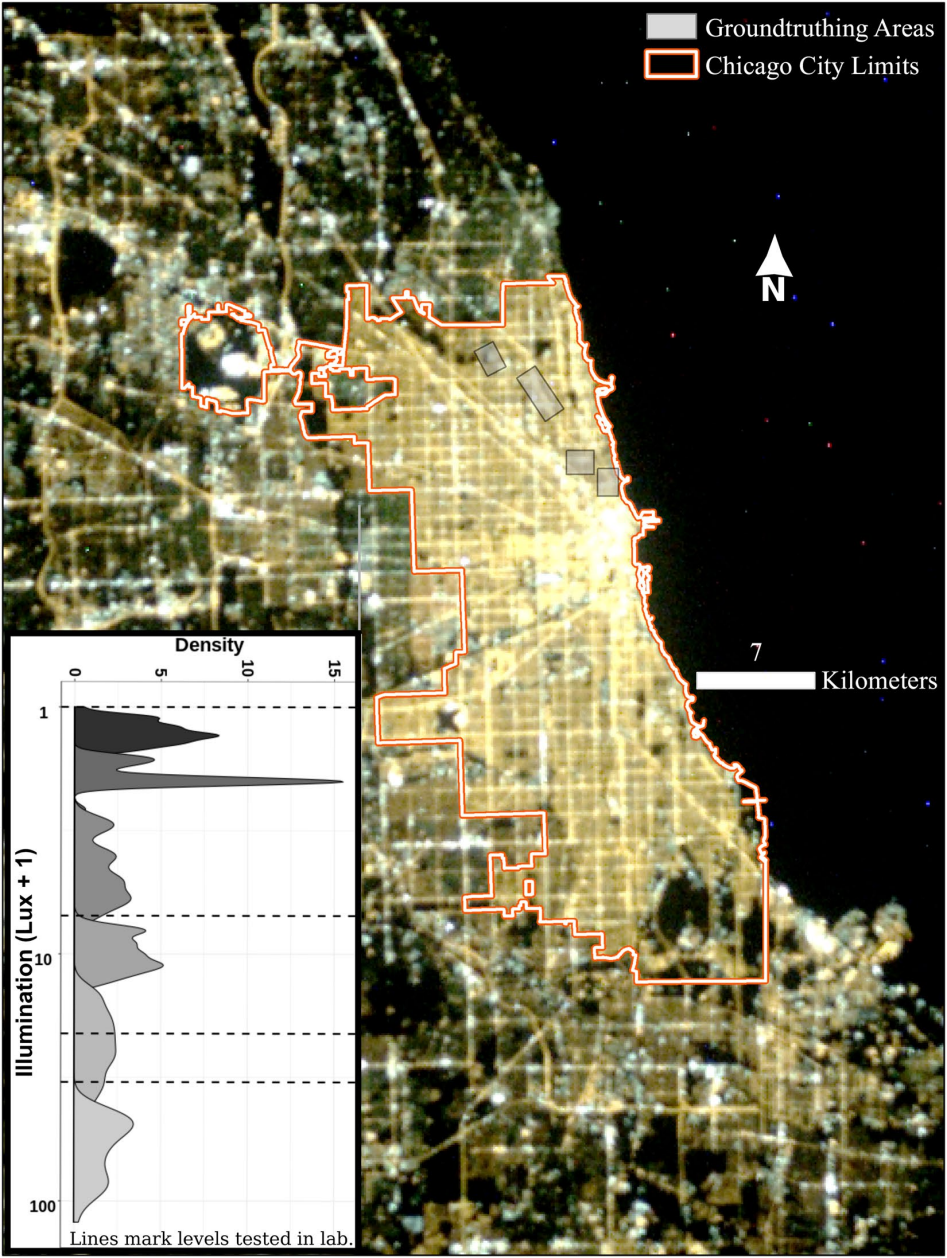
The intensity of light pollution varies across the city of Chicago. A 2013 nighttime image from the International Space Station (ISS) shows concentrations of nighttime lights near the central business district and along major roadways, as well as in large parking lots. The inset shows the distribution of clusters of control points’ illumination, sampled in areas of moderate and diverse light intensity shown on the satellite image, identified using finite mixture models. Laboratory illumination levels were chosen to be broadly representative of the most common varying light levels measured in the control areas.

We combined nighttime images from the International Space Station (ISS) and ground-level illumination measurements (in lux) to describe the levels of light at night throughout Chicago. Illumination measurements (N = 998) were collected approximately 1.5 meters above the ground at control points around the city (see Fig. 1), with measured values ranging from ≤ 0.01 to 121 lux. To identify commonly occurring light levels, we used finite mixture models to cluster illumination values across our control points. We used these clusters as reference points to select illumination levels to test in our laboratory experiments. The optimally fitting finite mixture model, selected using Bayesian Information Criteria (BIC), identified six illumination clusters (Fig. 1 inset), with median illumination values of 0.3, 1, 4, 9, 22, and 56.5 lux and maximum illumination values of 0.5, 1.6, 6, 12.7, 42, and 121 lux.

### Typical nighttime light levels in Chicago alter mammalian locomotor behavior

**Figure 2:**
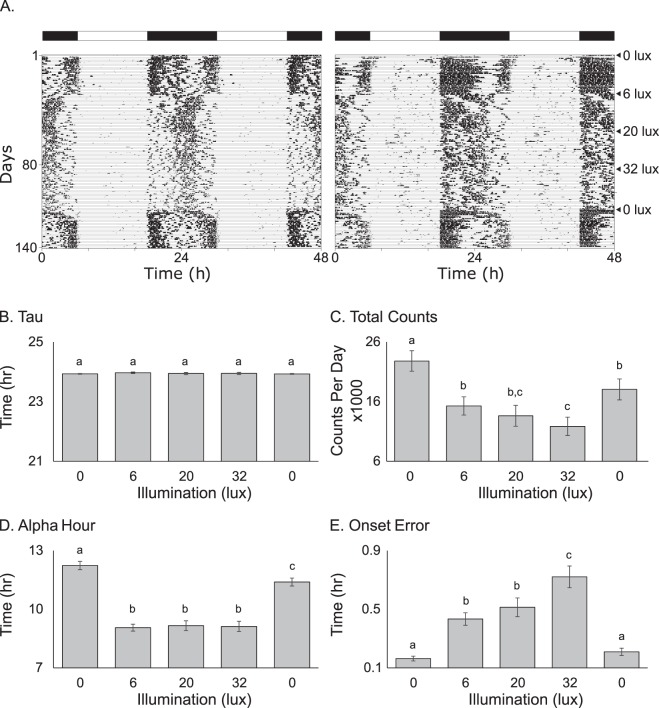
Increasing levels of nocturnal light significantly change mammalian locomotor behavior. Double plotted actograms of two male mice (**A**) clearly demonstrate that increasing levels of nocturnal light change overall activity patterns in these animals. Time on the x-axis represents local time and the light/dark bar at the top of each actogram corresponds to the light (white) and dark (black) phases of the light cycle. Levels of light during the dark phase (black bars) were systematically increased every 4 weeks as depicted on the right of panel A. No significant differences were found between male and female animals (n = 8 and 7, respectively). Quantification of these patterns demonstrate that while the period (Tau; **B**) of locomotor activity is unchanged, the total counts (**C**) and the length of the active phase (Alpha hour; **D**) significantly decrease and onset error (**E**) significantly increases with increasing levels of nocturnal light. When animals were returned to < 0.01 lux at the end of the experiment all values trended back toward values seen in the initial < 0.01 lux condition. All values in panels B–D are represented as mean ± SEM. Different letters above each bar represent significant differences with p < 0.05.

To simulate the effects of the Chicago lightscape on C57BL/6 J mouse models, we tested illumination at 6 lux, the maximum value of the low-illumination clusters; 20 lux, near the median value of the second highest illumination cluster; and 32 lux, which was at the upper 90th percentile of illumination levels observed in the control points (dashed lines; Fig. 1 inset). Figure 2A depicts example actograms of two male mice exposed to four consecutive weeks each at illumination levels of < 0.01, 6, 20, 32, and < 0.01 lux at night, respectively. Clear changes in the amplitude, duration, and timing of wheel running activity are evident for all levels of nighttime illumination. Analyses of the actograms from all animals (N = 15; Fig. 2B–E) demonstrated that the chi-square periodogram (23.95 ± 0.015 hours) was unchanged across light conditions, suggesting all animals remained entrained (circadian oscillators remained synchronized) to the light-dark (LD) cycle throughout the experiment.

Despite maintaining entrainment, animals’ activity levels and active phase length (alpha hour) decreased significantly with the introduction of nighttime illumination (overall effect for total activity χ ^2^ = 55.83, p < 2.17e-11; overall effect for alpha hour χ^2^ = 111.53, p < 2.2e-16; Fig. 2C,D). Total activity progressively decreased as light levels increased, with significant differences existing between the < 0.01, 6, and 32 lux groups (0.0053 < p < 2e-16; no significant differences were found between the 6 and 20 lux conditions). The length of the active phase significantly compressed from 12.24 ± 0.21 hours under < 0.01 lux conditions to 9.06 ± 0.18 under 6 lux conditions (p < 2e-16). No significant changes to the length of the active phase were found as illumination levels continued to increase past 6 lux (20 lux = 9.16 ± 0.25, 30 lux = 9.12 ± 0.26).

Exposure to dim nighttime illumination also significantly influenced activity onset timing precision (onset error; χ^2^ = 54.55, p < 4.02e-11, Fig. 2E). As nighttime illumination increased, onset error also increased from 0.22 ± 0.04 hours at < 0.01 lux to 0.72 ± 0.074 hours at 32 lux. Tukey-Kramer post hoc analysis revealed significant differences between the < 0.01, 6, and 32 lux groups (1.9e-13 < p < 0.0075), while no significant differences were found between the 6 and 20 lux conditions (Fig. 2E).

On concluding the light manipulations, all animals were returned to a final, four-week, LD cycle. No changes were observed in the periodogram under these conditions. The mean activity increased to levels higher than those at 20 lux (p < 0.0053) but consistent with levels seen at 6 lux (p < 0.12; Fig. 2C). The mean alpha hour expanded to 11.39 ± 0.201 hours; longer than the active phase under dimly-lit nights, while shorter than the active phase observed in the initial LD conditions (7.44e-5 < p < 3.44e-14; Fig. 2D). The mean onset error recovered completely to levels consistent with the initial < 0.01 lux conditions (Fig. 2E).

### Field measurements of mammalian activity show significant circadian changes similar to those found in the laboratory

To test the applicability of our laboratory findings in the field, we used camera trap data from the Chicagoland region to test the hypothesis that increased light levels would correspond to changed activity patterns, as seen in our laboratory experiments. To estimate artificial nighttime light levels animals might be exposed to around each camera trap, we used regression tree models calibrated by predicting our control points using two georeferenced ISS nighttime photography images. We present estimated illumination based on our best regression tree model for the Chicago city limits in Fig. S2. As the histogram indicates, illumination varies substantially. It is highest near the central business district, the city’s primary commercial streets, and around large parking lots.

Because our estimated illumination levels reflect only horizontal illumination, and to better approximate an animal roaming through the urban environment, we identified estimated illumination levels (in lux) for 1,000 randomly sampled points outside building footprints within 300 meters, 500 meters, and 1 kilometer of each camera, computing the mean estimated illumination for each buffer distance and illumination level dividing points using our best-fitting regression tree model. Using minimum BIC, we found optimal fit for logistic regression models dividing cameras at 6 lux within a 1 kilometer buffer for both diurnal and nocturnal species. To be clear, this does not imply that there are no detectable differences below this level; simply that this model is the most parsimonious way of explaining variation in species occurrence of the configurations estimated.

**Figure 3:**
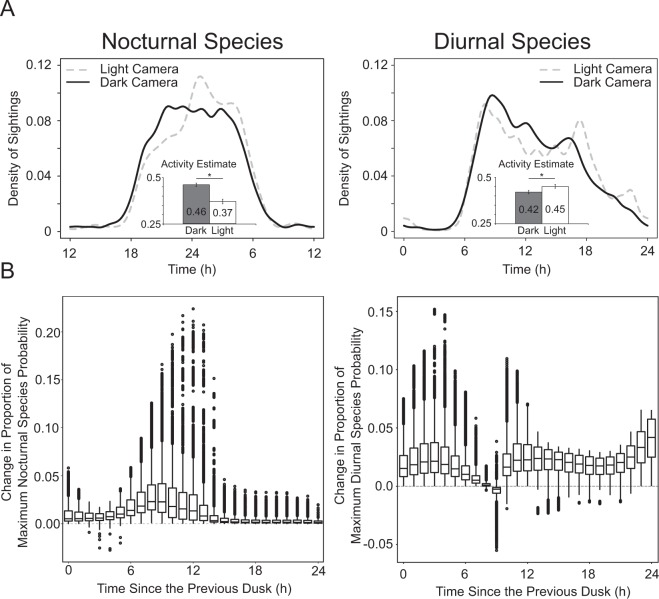
Populations of mammalian urban wildlife show significant changes in activity based on the level of light in their local environment. Daily activity profiles for nocturnal and diurnal mammals derived from tagged camera trap observations plotted based on local time (**A**). Activity profiles in light locations (locations with illumination > 6 lux) are represented as a dashed grey line while dark locations are represented as a solid black line. Significant differences were found between light and dark camera locations for both nocturnal and diurnal species. Panel A inset bar graphs represent overall activity estimates derived from the camera trap data. Inset values are represented as the mean ± SEM. Significant differences (p < 0.05; represented by an asterisk) were found between light and dark activity estimated for both groups of species. Logistic regression models (**B**) were used to control for temporal patterns of human activity and urbanization and show similar patterns to the raw density sightings in Panel A. (**B**) Simulates the predicted probability of observing nocturnal and diurnal species, sets each camera trap to light and then to dark, divides these values by the maximum probability for each condition, and then subtracts the value for simulated light cameras from simulated dark cameras. Horizontal lines in each box represent median values and the top and bottom of each box represent the 75th and 25th percent of each value. Single dots are values beyond 1.5X the interquartile range. The distribution of the boxplots highlights periods when species’ activity around cameras is expected to be most different between dark and light conditions.

We then used the “activity” package in R to fit kernel density functions to the camera trap data and then compared these functions to determine differences in activity distribution across light and dark cameras, grouped according to our best-fitting logistic regression models. Because there are many possible zeitgebers (environmental signals affecting animals’ entrainment) in a field setting, and because we have observations throughout the year, we decided to conduct these analyses using clock time. Figure 3A depicts the density of animal sightings in light (gray dashed line) and dark (black line) regions for 5 species of nocturnal (cat, coyote, opossum, raccoon, and rat; Light N = 1980; Dark N = 6772) and 3 species of diurnal (dog, fox squirrel, and grey squirrel; Light N = 15,397; Dark N = 12,453) mammals across a three year period.

We found significant differences between the activity distribution in light and dark camera locations for nocturnal species (Kolmogorov-Smirnov; D = 0.55, p ≤ 2.2e-16). Nocturnal density plots (Fig. 3A) exhibit a slight delay in the onset of activity in light camera locations and a compression of activity into the early morning hours. Nocturnal species demonstrated 19.6% higher activity levels in dark camera locations compared to light (Wald test; w = 32.96; p = 1.28e-8; Fig. 3A inset).

Diurnal species’ density plots also showed significant differences between activity distributions in light and dark camera locations (Kolmogorov-Smirnov; D = 0.08, p ≤ 2.2e-16), with a pattern opposite that of the nocturnal species. Diurnal species’ activity onsets are similar, but offsets are delayed. Diurnal species demonstrated overall activity 6% lower in dark camera locations, as compared to light camera locations (Fig. 3A; Wald test; w = 4.17; p ≤ 0.0412).

Some of the differences seen in urban wildlife species in light and dark areas could be attributed to different patterns of human activity and urbanization at the various camera locations. In addition, as mentioned above, there are several possible zeitgebers in a field setting, and controlling for these factors might affect our results. As explained in the methods, we used logistic regression observation probability models to control for these possible confounding variables, estimating models for over 250 combinations of illumination level divisions, buffer distances, species activity onset time, and functional forms, selecting the models with minimum BIC for nocturnal and diurnal species (see Table S1 for a summary of controls and Table S2 for coefficient estimates and goodness-of-fit measures and Fig. S3 for predicted probabilities of our primary variables of interest). To make it clear where the shapes of the activity curves differ for light and dark cameras, we estimated, for each model, the predicted probability for all observations when illumination values were set to the low illumination condition (≤ 6 lux), then the predicted probability when they were set to the high illumination condition (> 6 lux), with all other variables for each observation kept the same. We then divided the predicted probabilities in each condition by the maximum predicted probability for any camera-hour in that condition, thereby normalizing for the different levels of species abundance near each camera, which could drive differences in absolute predicted probabilities. Finally, we subtracted the normalized value for cameras when the illumination condition was assigned to low illumination from the normalized value for cameras when assigned to the high illumination condition. Figure 3B presents boxplots showing the distribution of the results of this calculation, using time since the previous dusk, in hours to approximate the zeitgeber of interest. These boxplots highlight the hours at which the probability of observing a diurnal or nocturnal species, relative to the maximum probability of observation in that condition, are most different for cameras in light and dark areas.

Figure 3B makes clear that cameras in brighter areas have proportionally higher peak probabilities for nocturnal species later at night, as compared to dark areas. This pattern is similar to the clock time density analysis and is consistent with shifted peak activity later in the night, controlling for the traps’ geographic characteristics. For diurnal species, activity levels decline more slowly in the evening in lighter areas, resulting in a rather flat curve for these areas, so relatively higher activity levels persist later into the night than in the darker areas, and lighted areas have a more pronounced peak as dusk approaches.

### Photopollution fragments greenspace in Chicago

**Figure 4:**
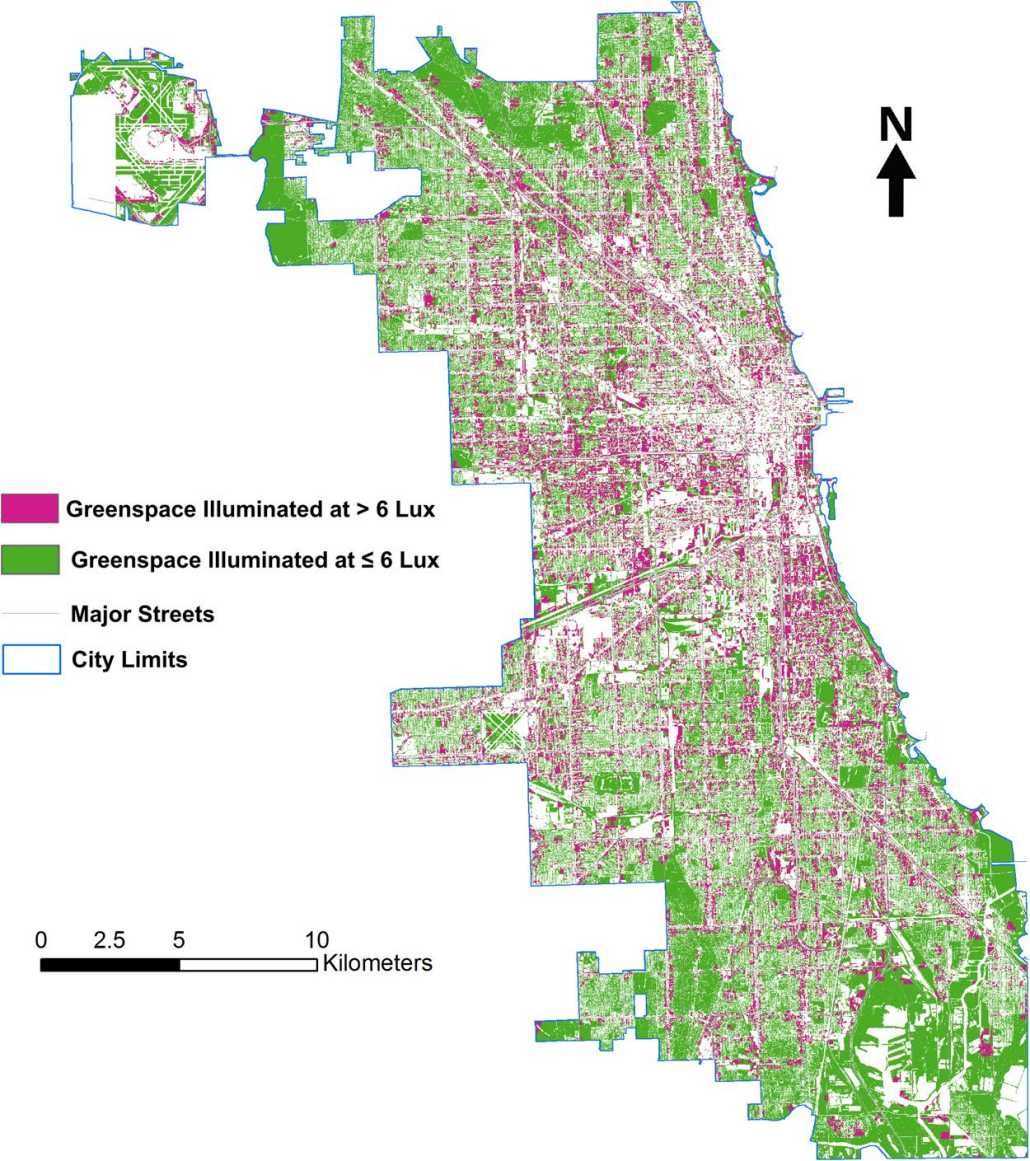
Behaviorally relevant levels of photopollution are found across the city and many smaller greenspace patches are completely affected by behaviorally relevant illumination levels. This map combines data from the city of Chicago showing urban greenspaces with areas estimated to be ≥ 6 lux, based on regression tree models fit on ground-level control points using ISS imagery.

To demonstrate the significance of these findings for urban greenspace planning, we used the regression tree models mentioned above to estimate illumination across the city. We considered greenspace as habitat for domestic, feral, and urban tolerant mammals. We mapped areas with illumination > 6 lux, as we found this value to be significant in both the lab and the field, but this should not be taken to imply that areas with less illumination are not photopolluted (Fig. 4). Rather, these maps are intended to demonstrate that photopollution identifies a significant source of degradation in urban greenspace. Greenspace illuminated at an estimated level greater than 6 lux follows major roads and activity centers, such as universities, parks, etc. Smaller greenspace patches around Chicago are also heavily affected (Fig. 4). The percentage of greenspace degradation varies substantially across official neighborhoods (Fig. S4). We estimate approximately 36% of the total greenspace in Chicago experiences nighttime light levels > 6 lux, and 45% of Chicago’s official neighborhoods experience photopollution across at least half their greenspace, ranging from 5% to 98%, with an average of 48%. The most highly photopolluted neighborhoods, with over 90% of their greenspace affected, are found around the central business district.

**Figure 5:**
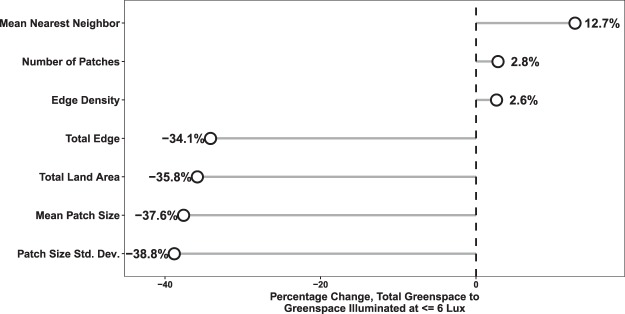
Chicago’s greenspace illuminated at over 6 lux is substantially different in extent and spatial structure from its total greenspace. This graphic presents percent changes in a range of landscape metrics between the entire greenspace and greenspaces estimated to be lit at above 6 lux. The metrics are based on patches, which are blocks of continuous greenspace cover. Over one-third of the greenspace areas in Chicago are exposed to illumination levels > 6 lux, and light-exposed areas split large greenspaces patches, resulting in substantial reductions in the mean patch size. At the same time, small greenspace patches tend to be completely exposed to behaviorally relevant artificial light levels, resulting in only small increases in the total number of patches. The splitting of the largest and elimination of the smallest patches further results in substantial declines in the standard deviation of patch size, a decline in the total edge of all patches, and a moderate increase in the mean distance between patches and their nearest neighboring patch.

We quantified these landscape changes using several landscape pattern metrics (Table S3 and Fig. 5). Based on the landscape metrics presented in Fig. 5, greenspaces illuminated at or below 6 lux have lower average patch size than greenspace as a whole, as well as lower total edge, slightly higher mean nearest neighbor distance, and, interestingly, a substantially lower standard deviation of patch size. These findings indicate the primary impacts of light pollution on open space in the city are, first, to significantly modify small patches, and, second, to divide large patches, resulting in a matrix composed primarily of separated patches of moderate size. To be clear, we do not mean by this analysis to imply that illuminated greenspaces have no conservation value but, rather, that not only the presence of illumination, but also its spatial pattern, can change the way we think about urban habitat.

## Discussion

Our study provides extensive new evidence that anthropogenic changes to nocturnal illumination can affect behavioral patterns of nocturnal and diurnal species in the laboratory with parallel associations in the field. We use a highly cost-effective and widely applicable suite of methods that could be adopted for other studies or planning purposes without requiring substantial financial resources. By reproducing a representative simulation of urban lightscapes in a laboratory and then comparing behavioral changes observed in the laboratory setting to camera trap data, we demonstrate that even relatively low levels of nocturnal illumination can be associated with deformation in species’ activity levels and patterns. Nocturnal light tends to associate with compressed activity patterns and activity shifts later in the evening for both nocturnal laboratory animals and urban wildlife. As seen in Fig. 3, the consistency between our laboratory and field findings, which hold when controlling for measures of human activity, land cover, and land use in areas surrounding our camera sites, suggest that urban animals’ behaviors respond to urban lighting regimes. It seems unlikely such strikingly similar findings in the two settings occurred by accident.

Our findings also corroborate previous literature. It is well known that light duration and intensity have opposite effects on the activity of most nocturnal and diurnal species^36–39^. Aschoff^36,37^ demonstrated that nocturnal species decrease activity in constant light while diurnal species increase activity. Garber^38^ used the term “night light niche” to describe the extension of diurnal species activity near artificial lighting at night. More recent studies, using similar conditions of altered nighttime light levels, also demonstrated that nighttime light decreases overall activity and changes the duration of the active phase^40–47^.

The most prominent circadian effects found in the lab were the significant decrease in activity levels, shortening of the active phase, and the increase of the onset error. Such changes suggest alterations to the circadian waveform and pacemaker function. Though further experiments will be required to determine the exact mechanisms underlying these changes, the fact remains that while the laboratory animals were able to maintain entrainment, confining their activity to the dim light phases, their overall behavior (and physiology) changed substantially after release into dark cycles ≥ 6 lux. These changes could lead to a number of other adverse effects, including changes in weight, metabolism, immune function, cognition, anxiety and affective responses^48–54^. In addition, increased nighttime light could lead to altered stable states of an organism’s circadian system. These states would be expected to vary from organism to organism, thereby altering the structure, composition, and interactions within the temporal niches of photopolluted environments^55^. Such changes would not only be detrimental to urban niches and wildlife but also to the many human inhabitants of these same environments.

Our work advances existing literature by providing a low-cost way to connect laboratory and field research. Field illuminance measurements like the ones described here are difficult to perform, especially in urban environments, due to the masking effects of trees and buildings^56,57^. We measured the levels of nighttime light in the laboratory and field using horizontal illuminance measured in lux. While this is an extremely common metric used in chronobiological and ecological laboratory and field studies^44,47,49,58–62^, a variety of other techniques and metrics exist^63^. Each technique comes with particular limitations when measuring levels of nighttime light in complex field environments^63^. For example, calibrated digital cameras equipped with a fish-eye lens have been used to measure scalar illumination^60^. While scalar illumination would be a better metric of actual exposure^60,63^, equipment for such measurements is expensive or requires difficult calibration steps, precise orientation of the detector, and appropriate conditions to minimize vignetting and distortion^63^. Modelling characteristics of areas around the traps, with trap observations as dependent variables, however, mitigates–though does not eliminate–concerns about exposure levels, an important challenge in photopollution field studies^64^. Furthermore, one of the goals of our study was to develop an easily accessible technique, using common equipment that could be transferred to other urban and semi-urban environments. The wide and proficient use of low cost “Sky Quality Meters”^56,60^ made that approach particularly attractive, due to its potential for widespread replication. As technologies and techniques for measuring scalar illumination become more widely used, our technique could be easily modified to accommodate these new data.

We measured illuminance in lux (as opposed to irradiance) because of its widespread use and accessibility^44,65^. The unit of lux is also beneficial because it is a photobiological quantity (derived from the SI unit Candela) used as a measure of perceived light intensity by the human eye, making it extremely appropriate for biological studies such as the ones described here^66^. However, it should be noted that humans’ light sensitivity differs from the diverse group of mammalian taxa studied, especially nocturnal species adapted to very low light environments. Lux also does not take into account spectral sensitivity, which differs across the species studied and is an important component of how species respond to illuminated environments^67^. Despite these differences in perceptual and spectral sensitivity, however, our data (combining several species) strongly demonstrate the broad impact of nighttime illumination on both nocturnal and diurnal animals. Future work focusing on specific species would be extremely beneficial, especially for species of concern to conservationists^68^.

Our relatively low-cost method further demonstrates how remote sensing can support conservation^69^. Although remote sensor data on artificial light has been used to model species distributions at larger scales^34,35^, ISS imagery provides a higher-resolution alternative at the urban scale. Researchers could replicate these techniques in sites with extant camera trap data to test these findings’ generalizability.

As urban areas continue to expand and an increasing number of wildlife species encounter human-modified landscapes, lighting regimes have the potential to significantly alter the functional extent and connectivity of urban habitats. Recent work suggests planners must move beyond a binary division between patches of habitat and a broader, inhospitable urban matrix, thinking instead of the large range of ways in which urban microhabitats have differential effects on species^70–73^. Similarly, Rich and Longcore^65^ suggest that professional conservationists also need to recognize the impact of light pollution as an important variable when protecting and preserving urban environments. If even low nocturnal light exposure can result in behavioral disturbances, the conservation value of extant urban greenspace may be lower – or at least different - than expected or hoped. As can be seen in Fig. 4, greenspace in Chicago illuminated to different degrees can have a meaningfully different geography than greenspace considered as a whole. While we do not assess the impacts of these patterns, changes in habitat amount and fragmentation are known to affect biodiversity at the landscape scale^74^. Alteration of small patches particularly can be a problem when patch size variation is high or small patches dominate^75–77^, features that are likely quite common in urban environments. To the extent that nocturnal illumination affects species’ behaviors, this may affect how we should be thinking about habitat fragmentation.

Because of these potential implications for how we understand habitat in urban environments, our findings not only provide further information about the potential behavioral effects of nocturnal illumination, they also have some important implications for urban design and policy, supporting calls to include analysis of artificial lighting in landscape ecology studies^33^. The intensity of light pollution is affected not only by population density, but also urban form, economic development, commuting patterns, and local policies^30,78,79^. Local governments have a variety of policy tools at their disposal to mitigate light pollution, which can be incorporated into zoning regulations or building codes. The most effective options likely include increasing the extent of unlit natural and seminatural areas, and reducing “trespass” of lighting into unneeded areas^78^. Planners should take heed of Lacoeuilhe, *et al*.’s^80^ advice to consider not only the physical network of patches and corridors defining urban greenspace, but also the “nocturnal network” formed by relatively dark areas, in order to best support urban wildlife.

## Materials and Methods

### Nighttime images

For this study, we acquired multiple ISS images taken on January 19, 2008 (ISS016E024220), January 31, 2012 (ISS030E061820) and October 9, 2013 (ISS037E008303). We manually georeferenced the ISS images by matching control points to a base map at identifiable locations, primarily street intersections, corners of buildings, parks, airports, and other visible features. Using the control points, the images were georeferenced to the State Plane Coordinate System (NAD 1983 StatePlane Illinois East FIPS 1201 Feet), with RMSEs approaching 0.

### Control point collection

In order to use ISS images to predict ground-level illumination values, we collected 998 valid control point measurements from transects in the sampling areas seen in Fig. 1. We measured horizontal illumination using a Gossen Mavolux 5032B (www.gossenmetrawattusa.com; dynamic range 0.01:199,900 lux), with the receptor held approximately 1.5 meters above the ground. Sample locations were recorded using a DeLorme Earthmate PN-60 (mapstore.delorme.com) GPS unit.

To identify typical illumination across the city, which could delineate different artificial light environments, we used Gaussian finite mixture model clustering, as implemented in the mclust package^81,82^ in R 3.4.1^83^. This approach allows for the use of standard model selection measures to identify a set of clusters optimizing the tradeoff between fit and model complexity. Using Bayesian Information Criteria (BIC) for model selection, we identified six clusters of illumination values from the sampled points, the distribution of which are presented in Fig. 1. We then used summary statistics of the illumination of each cluster to help select illumination values to test in our laboratory experiments. Visualizations for these data utilized the R package ggplot2^84^.

### Circadian behavioral testing

We individually housed male and female C57BL/6J mice (N = 8 and 7, respectively) in a ventilated, light-tight cabinet with free access to food and water in cages that contained a running wheel. White LED lights mounted above the cages provided 12 h of daytime light at a level of 1800 lux, and 12 h of nighttime light that ranged from < 0.01–32 lux. Daytime light intensity and duration remained constant for the duration of the experiment, while nighttime light levels increased every 4 weeks to study < 0.01, 6, 20, 32, and < 0.01 lux, respectively. We maintained the duration and intensity of the light using the Clocklab (Actimetrics) program. This software also continuously recorded wheel running activity for all animals. Clocklab data analysis software quantified the activity data from the final ten days of each light condition to measure the chi-square periodogram, total activity, length of active phase, and onset error for each animal. The mean ± SEM are reported for each parameter. We analyzed the behavioral data using R 3.3.0^83^, testing for normality using a Shapiro-Wilks test and comparing individual groups using linear mixed models (lme4 package in R^85^) followed by Tukey-Kramer post-hoc tests. In all cases, we treated the experimental animals with the appropriate concerns and operated in accordance with all applicable ethical and animal care guidelines. The NEIU Institutional Animal Care and Use Committee approved all laboratory animal work (protocol #AS-0516-0519-01).

### Geospatial modeling

Pixel values from the ISS image are not on a behaviorally meaningful scale and, while at a higher resolution than most nighttime light satellite datasets, are still at a relatively coarse resolution for the analysis undertaken here (Fig. 1). To resolve these two problems, we used random forest regression, as implemented in the randomForest package^86^ in R 3.4.1^83^ to predict the illumination values measured at our control points, averaged within a 20-meter radius, using the pixel values of the first band of the 2008 and all bands of the 2013 ISS images. We fit the random forest regression models using the pixel values of each of these bands at different distances. First, we used the value of the pixel in which each control point was located and then computed the weighted mean pixel value within 5, 10, and 20 meters, with the weight defined by the proportion of the area within these buffers falling in each pixel. Using these values as prediction variables, we predicted the natural logarithm of the mean illumination within 20 meters of each control point, plus 1. We fit the model on 798 control points, saving 200 for cross-validation. The optimal model, identified using the tuning function in the randomForest package, explained 49% of total variance in the outcome variable and had no lower than 60% accuracy in classifying control points as above or below specific lux values in the cross validation sample (Fig. S5).

Using the fitted random forest model and the ISS images, we predicted illumination values at 2,643,771 points across the smallest ISS image extent. To ensure coverage, 2,000,000 of these points were generated randomly, while the remaining 643,771 points were generated on an evenly spaced grid. We converted the points’ estimated illumination to a raster layer with a 30-meter resolution by taking the average illumination values of predicted points within each raster cell, producing an estimated light intensity map (Fig. S2).

### Field validation of activity changes

We derived wildlife monitoring data from a long-term study of urban mammals in the Chicago region^87,88^. In brief, data were collected from over 100 locations (83 used for this study and 62 with complete data for logistic regression modelling) using trail cameras deployed 4 times per year for approximately 28 days each. Sites were located along three urban to rural gradients that initiated in downtown Chicago and travelled 50 km to the west, northwest, and southwest. Data for this study came from years 2011–2013.

We analyzed a total of 347,528 species sightings from 83 cameras, selected based on their proximity to the city (the majority falling within 25 km of the Chicago Loop) and estimated light environment (based on the geospatial models described above), for activity pattern differences between light and dark locations. Cameras were separated by at least 1 kilometer to minimize spatial autocorrelation^87,88^. While our limited research budget of $6,000 was insufficient to study diurnal species in the lab, the camera traps observed both nocturnal and diurnal species, allowing us to analyze both groups. Our goal was to empirically identify behaviorally meaningful differences in illumination levels across areas through which the observed species roam. To do this, we estimated average illumination within 300 meter, 500 meter, and 1 kilometer buffers around each camera by taking 1,000 sample points outside building footprints, using the regression tree model described above to estimate illumination at these points, and then computed the mean value for each camera trap. We then estimated 270 logistic regression models, with the presence of nocturnal or diurnal species during each hour the camera traps were active as the dependent variables, using a combination of cutoff points to divide light from dark cameras (1, 3, and 6 lux) within each buffer distance, interacting these values with several functional forms for activity rates across each dusk-to-dusk period, measured in radians, calculated using the suncalc^89^ package in R. We used minimum BIC to select the combination of terms optimizing model fit for nocturnal and diurnal species to select appropriate buffer distances and illumination levels. For both diurnal and nocturnal species, our optimal model divided cameras at 6 lux within 1 kilometer.

We used this value to divide the cameras into light (> 6 lux, N = 29) or dark ( ≤ 6 lux, N = 54) locations. Nine species were selected for analysis and divided based on their nocturnal (Cat N = 929, Coyote N = 780, Opossum N = 3313, Raccoon N = 3138, Rat N = 592, and Skunk N = 404) or diurnal (Dog N = 19083, Fox Squirrel N = 502, and Gray Squirrel N = 8265) activity patterns as captured by the camera traps. While coyotes are not typically characterized as nocturnal, they have been shown to become nocturnal in the presence of human activity^90,91^ and this is supported empirically by individual species’ activity profiles (Fig. S6). A circular kernel probability density function was used to calculate, visualize, and statistically compare nocturnal and diurnal species at each light level using the activity and overlap packages in R^92,93^. The data visualizations used the overlap package while the quantification of overall activity levels was done using the activity package. Overall activity distributions between light and dark cameras were compared using a Kolmogorov-Smirnov test, while activity level estimates were compared using a Wald test.

While the algorithms available in the activity and overlap packages are unidimensional, making it impossible to control for multiple zeitgebers, it is possible to control for human and other rhythms using our logistic regression models. This freed us to use time since the previous dusk, measured in radians and scaled to 2π for each dusk-to-dusk period, to model species’ activity distributions in high and low illumination settings. Because dusk is an imprecise indicator of species activity onset, we estimated models setting two, one, and zero hours before and after solar dusk to π. The optimal model for nocturnal species set two hours after solar dusk to π, while the optimal for diurnal species set two hours prior to dusk to π. For clarity and simplicity, we present our graphical results (utilizing the R packages ggplot2^84^ and visreg^94^) for these models in terms of hours since the previous dusk.

We used the logistic regression models to control for several confounding variables. As human social rhythms are likely to affect urban wildlife behaviors, we included the average number of humans observed by each camera for each hour of the day across each season in which the camera was active. We also controlled for a variety of land-use and land-cover features around the camera traps. These included the extent of residential, dense residential, commercial, and industrial land use, as well as the extent of various land covers within 1 kilometer of each camera trap. For land use, we used the Chicago Metropolitan Agency of Planning (CMAP) 2013 land use inventory^95^, which classifies areas as residential (including both dense and light residential), commercial, and industrial. We used CMAP’s 2010 high resolution (2 feet by 2 feet) land cover dataset^96^ to identify greenspace. This dataset was derived from LiDAR data and high resolution aerial photography using a “top-down” approach to preserve tree canopy cover. The dataset includes seven land cover categories, including tree canopy, grass/shrub, bare earth, water, buildings, roads, and other paved surfaces. We controlled for changes in species’ activity across the seasons of the year by including a quadratic term for the length of the day. We also controlled for the fraction of moon visible, computing both this and the previous measure using the suncalc package in R. Finally, we used the natural logarithm of the distance of the trap from the Chicago Loop to control for relative position in the city. Sources and more detailed explanations of the variables in our models are presented in Table S1, and our optimal estimated models are presented in Table S2.

To further evaluate the relationship between photopollution and wildlife habitat, we mapped the intersection between areas illuminated at greater than 6 lux and greenspace in Chicago by extracting tree canopy, grass/shrub, and bare soil covers as wildlife habitat proxies. Then we identified those open spaces with light levels > 6 lux, the level at which significant behavioral changes occurred in the lab and in the field, as affected by photopollution (Fig. 4). Due to the differences in spatial resolution between the land cover data and the light intensity layer, we resampled the land cover data to 30 meter by 30 meter resolution. Finally, we calculated several landscape metrics to quantify the degree of habitat fragmentation due to photopollution (see Table S3 for explanations) using FRAGSTATS 4.2^97^.

## Supplementary information

Supplemental Figures and Tables

Data and Replication Package

## Data Availability

All data, other than camera locations, and R code are available in the supplemental materials. R code has been updated to run on current package versions.
