# Supplementary material for: Mapping behaviorally relevant light pollution levels to improve urban habitat planning
Source: Sci Rep. 2019 Aug 15;9:11925. doi: 10.1038/s41598-019-48118-z (PMC6695421; doi:10.1038/s41598-019-48118-z)
Supplement: Supplementary file 1 — Supplemental Figures and Tables [file 41598_2019_48118_MOESM1_ESM.pdf]

Mapping behaviorally relevant light pollution levels to improve urban habitat planning

Aaron E. Schirmer[1*†], Caleb Gallemore[2†], Ting Liu[3†], Seth Magle[4], Elisabeth DiNello[1], Humerah Ahmed[1], Thomas Gilday[3]

Affiliations

[1] Northeastern Illinois University, Dept. of Biology, 5500 St. Louis Ave., Chicago IL 60625
[2] Lafayette College, International Affairs Program, 730 High St., Easton, PA 18042
[3] Northeastern Illinois University, Dept. of Geography and Environmental Studies, 5500 St. Louis Ave., Chicago IL 60625
[4] Lincoln Park Zoo, Urban Wildlife Institute, 2001 N Clark St, Chicago, IL 60614

*Correspondence to: a-schirmer@neiu.edu | (773)442-5617
†Authors contributed equally to the writing of this manuscript

Supplemental Figures and Tables

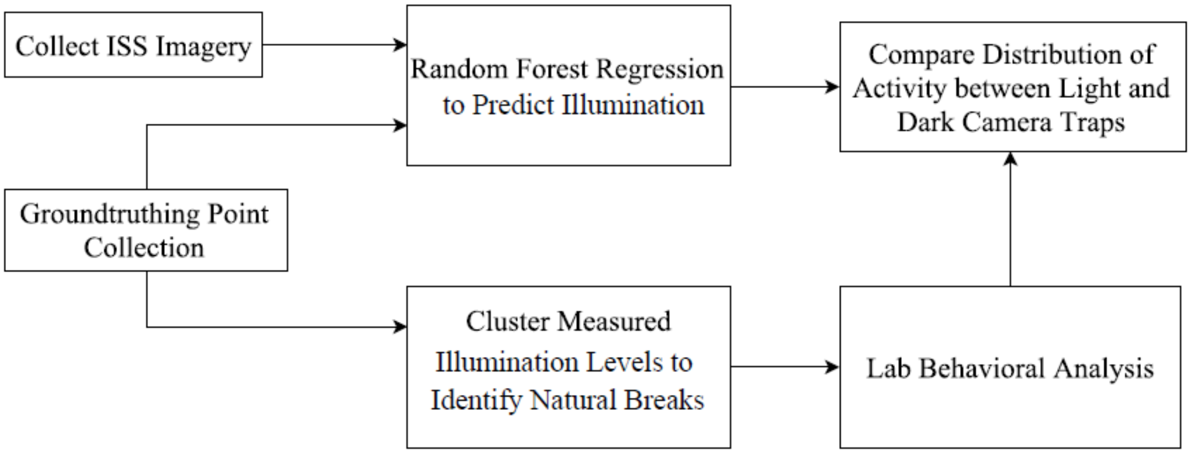

Figure S1. Schematic showing the research design flow, showing how outputs from one component of the research provided inputs for other components.

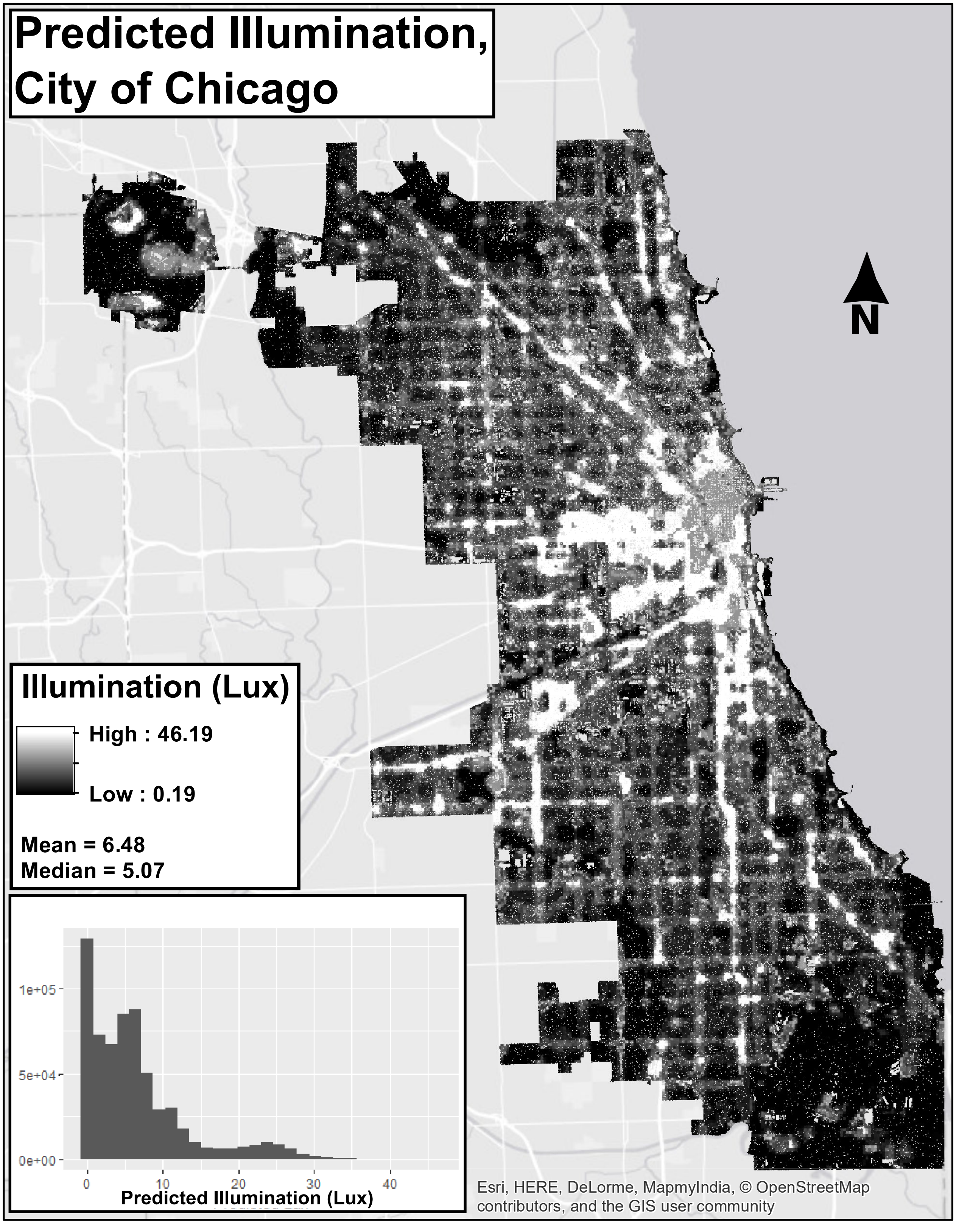

Figure S2. Predicted illumination levels for the city of Chicago, based on best fitting regression tree model fit on control points. Illumination represents predicted average levels in a 20-meter radius around each point. The histogram demonstrates that a substantial portion of the city area is estimated to be above the 6 lux threshold found to have behavior effects in our laboratory experiments.

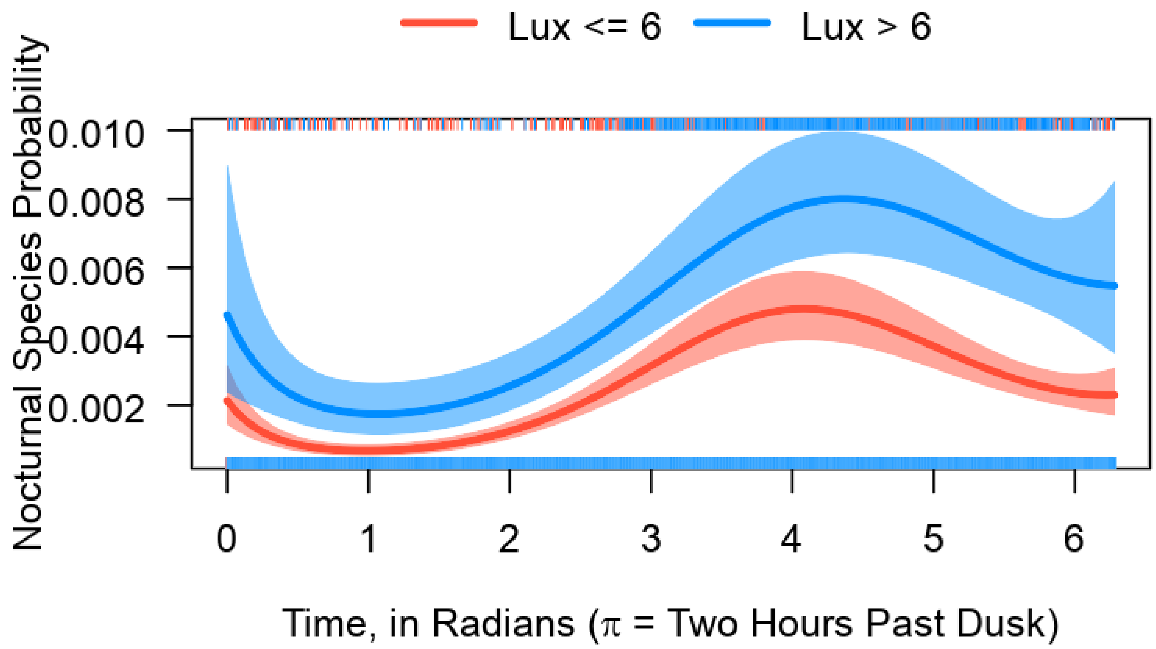

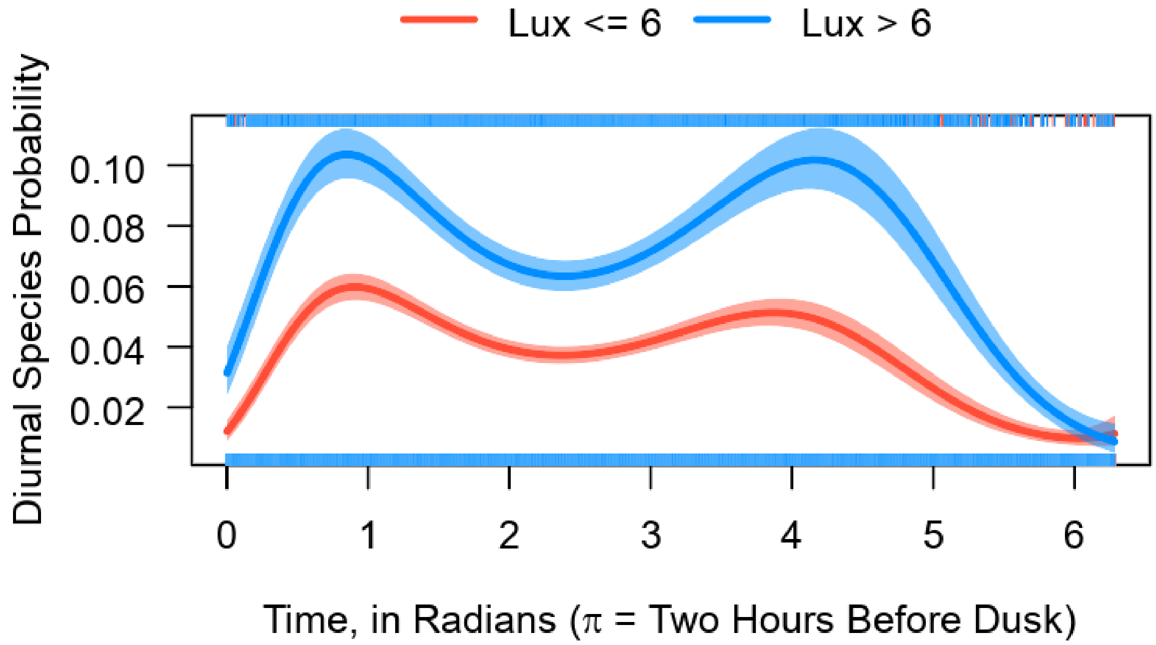

Figure S3. Predicted probabilities of observing nocturnal and diurnal species, by illumination. Computed from best-fitting models and visualized using the visreg package (Breheny & Burchett, 2017) in R. Confidence envelopes set at 95%.

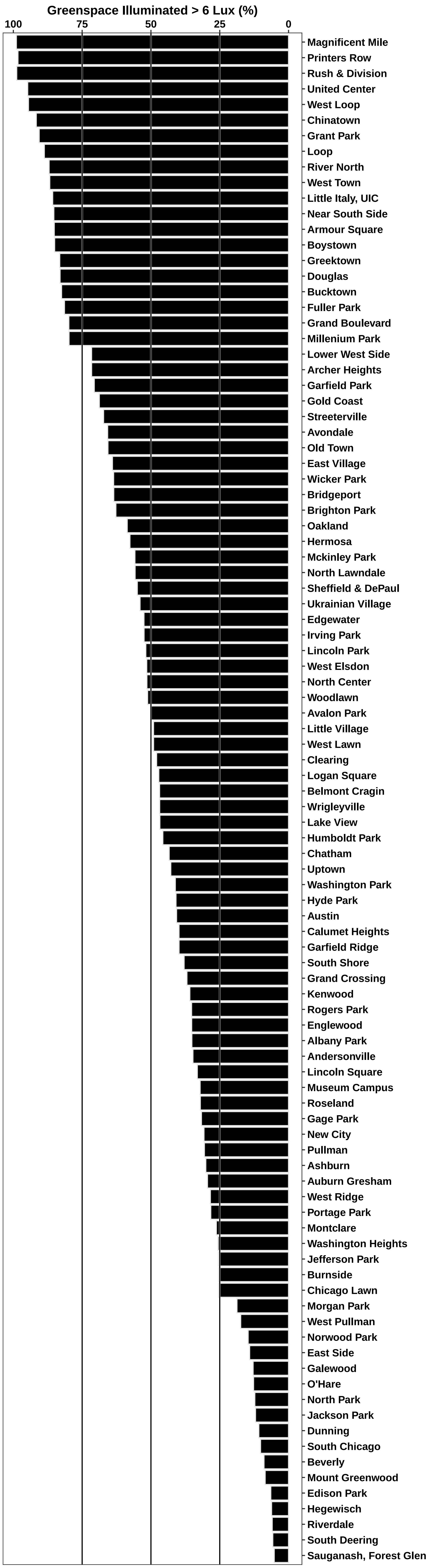

Greenspace Illuminated > 6 Lux (%)
100
75
50
25
0
Magnificent Mile
Printers Row
Rush & Division
United Center
West Loop
Chinatown
Grant Park
Loop
River North
West Town
Little Italy, UIC
Near South Side
Armour Square
Boystown
Greektown
Douglas
Bucktown
Fuller Park
Grand Boulevard
Millenium Park
Lower West Side
Archer Heights
Garfield Park
Gold Coast
Streeterville
Avondale
Old Town
East Village
Wicker Park
Bridgeport
Brighton Park
Oakland
Hermosa
Mckinley Park
North Lawndale
Sheffield & DePaul
Ukrainian Village
Edgewater
Irving Park
Lincoln Park
West Elsdon
North Center
Woodlawn
Avalon Park
Little Village
West Lawn
Clearing
Logan Square
Belmont Cragin
Wrigleyville
Lake View
Humboldt Park
Chatham
Uptown
Washington Park
Hyde Park
Austin
Calumet Heights
Garfield Ridge
South Shore
Grand Crossing
Kenwood
Rogers Park
Englewood
Albany Park
Andersonville
Lincoln Square
Museum Campus
Roseland
Gage Park
New City
Pullman
Ashburn
Auburn Gresham
West Ridge
Portage Park
Montclare
Washington Heights
Jefferson Park
Burnside
Chicago Lawn
Morgan Park
West Pullman
Norwood Park
East Side
Galewood
O'Hare
North Park
Jackson Park
Dunning
South Chicago
Beverly
Mount Greenwood
Edison Park
Hegewisch
Riverdale
South Deering
Sauganash, Forest Glen

Figure S4. Percentage of greenspace in Chicago estimated to be illuminated at > 6 lux, by neighborhood.

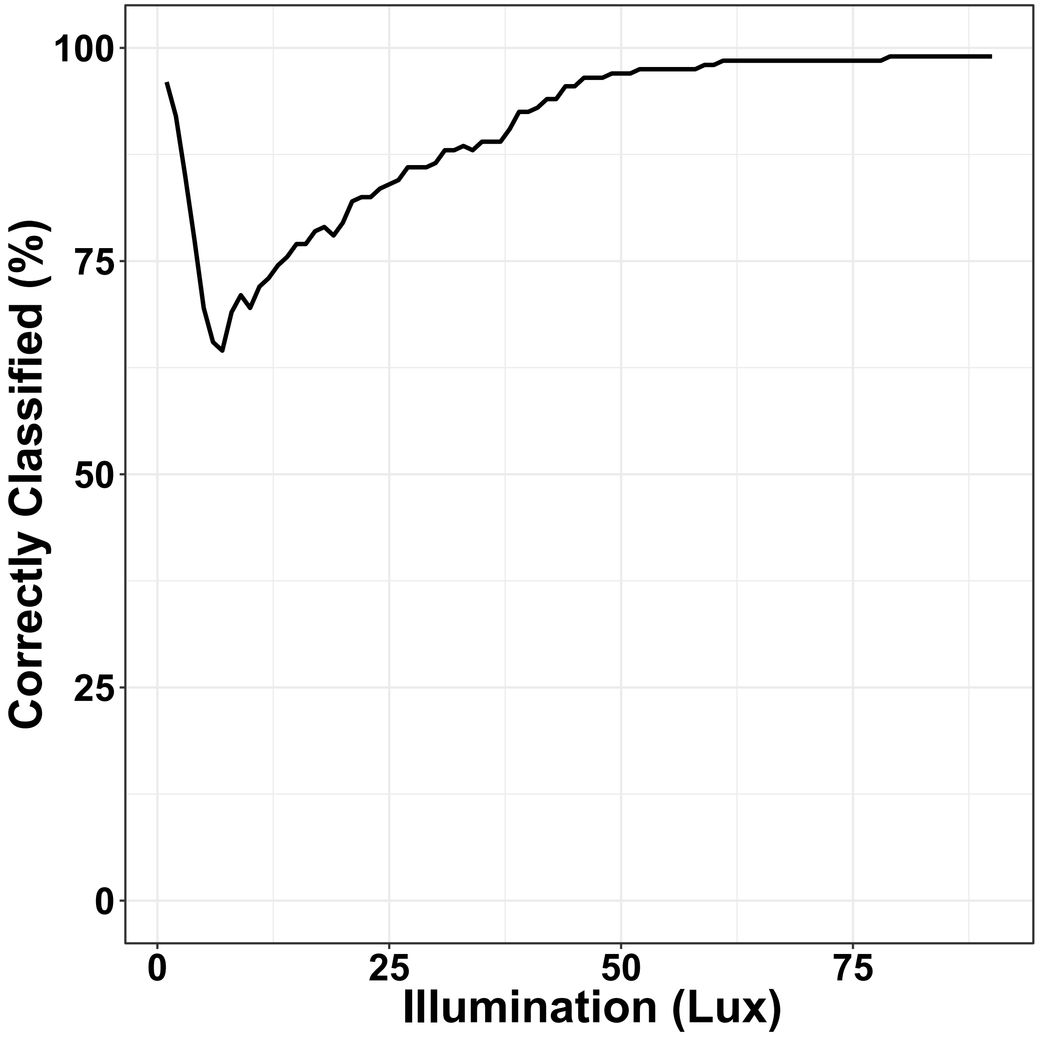

Figure S5. Percentage of cross-validation control points correctly classified as above or below a lux value for the range of illumination observed.

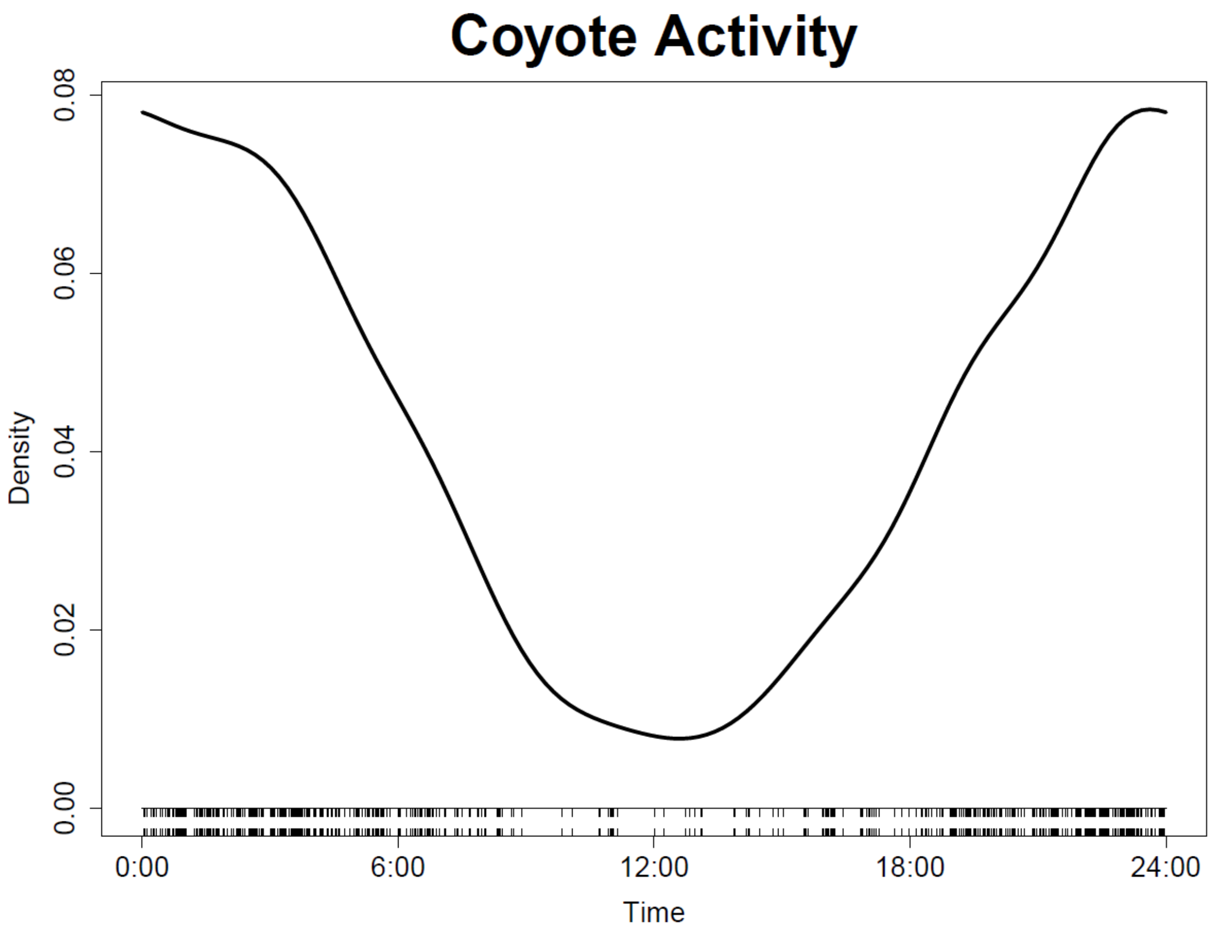

Figure S6. Daily activity profiles for Coyotes derived from tagged camera trap observations and plotted based on local time.

Table S1. Descriptions, sources, and summary statistics of variables used in logistic regression models.

Variable Type	Variable	Description	Source	Mean (Std. Dev.) [Min., Median, Max.]
**Dependent**	Diurnal Species Occurrence	Binary variable; 1 if a diurnal species (dog, fox squirrel, and grey squirrel) was observed by the camera during the hour and 0 otherwise	Camera trap records	0.042 (0.2) [0, 0, 1]
	Nocturnal Species Occurrence	Binary variable; 1 if a nocturnal species (cat, coyote, opossum, raccoon, or rat) was observed by the camera during the hour and 0 otherwise	Camera trap records	0.015 (0.12) [0, 0, 1]
**Independent**	High Illumination	Binary variable; camera traps with estimated mean lux values within 1 kilometer greater than 6	Authors' calculations based on predicted lux levels from random forest regression models	0.29 (0.45) [0, 0, 1]
	Time Since Dusk	Time since dusk, in radians; values are normalized so all dusk-to-dusk periods are 2π radians; for nocturnal species, π is one hour after solar dusk, while π is set to two hours prior to solar dusk for diurnal species	Authors' calculations using the suncalc package (Thieurmel & Elmarhraoui, 2019) in R.	3.14 (1.81) [0.0022, 3.14, 6.28]
**Human Activity Control**	Average Humans	Mean number of humans observed during the clock hour across the entire season, by camera	Camera trap records	0.56 (0.81) [0, 0, 6]
	ln(Distance from Loop)	Natural logarithm of the camera's great circle distance, in kilometers, from the Palmer House hotel in the Chicago Loop	Authors' calculations using fields package (Nychka, et al., 2015) in R.	2.46 (0.57) [0.7, 2.58, 3.18]
**Seasonal Control**	Length of Day	Time between dawn and dusk, in decimal hours	Authors' calculations using the suncalc package	12.47 (1.9) [9.43, 13.4, 15.9]

			(Thieurmel & Elmarhraoui, 2019) in R.	
**Zeitgeber Control**	Moonlight	Fraction of the moon illuminated, when visible; set to zero when moon is set	Authors' calculations using the suncalc package (Thieurmel & Elmarhraoui, 2019) in R.	0.011 (0.062) [0, 0, 0.47]
	Daylight	Binary variable; 1 if the time is after dawn and before dusk, 0 otherwise	Authors' calculations using the suncalc package (Thieurmel & Elmarhraoui, 2019) in R.	0.52 (0.5) [0, 0, 1]
**Local Environment Control**	ln(Light Residential)	Natural logarithm of one plus the total square meters of light residential land use within one kilometer	Authors' calculations using Chicago Metropolitan Agency for Planning (2015)	0.36 (1.88) [0, 0, 11.24]
	ln(Dense Residential)	Natural logarithm of one plus the total square meters of dense residential land use within one kilometer	Authors' calculations using Chicago Metropolitan Agency for Planning (2015)	13.8 (0.35) [12.91, 13.8, 14.42]
	ln(Commercial)	Natural logarithm of one plus the total square meters of commercial land use within one kilometer	Authors' calculations using Chicago Metropolitan Agency for Planning (2015)	12.69 (0.67) [10.17, 12.87, 13.06]
	ln(Industrial)	Natural logarithm of one plus the total square meters of industrial land use within one kilometer	Authors' calculations using Chicago Metropolitan Agency for Planning (2015)	11.8 (1.06) [2.8, 11.97, 13.71]
	ln(Tree Cover)	Natural logarithm of one plus the total square meters of tree cover within one kilometer as of 2010	Authors' calculations using Chicago Metropolitan Agency for Planning (2014)	7.58 (0.69) [5.13, 7.61, 8.94]
	ln(Grass)	Natural logarithm of one plus the total square meters of grass/shrub cover within one kilometer as of 2010	Authors' calculations using Chicago Metropolitan Agency for Planning (2014)	7.57 (0.8) [5.08, 7.85, 8.64]

	ln(Soil)	Natural logarithm of one plus the total square meters of bare soil within one kilometer as of 2010	Authors’ calculations using Chicago Metropolitan Agency for Planning (2014)	3.46 (2.86)<br>[0, 5.22, 8.69]
	ln(Water)	Natural logarithm of one plus the total square meters of water within one kilometer as of 2010	Authors’ calculations using Chicago Metropolitan Agency for Planning (2014)	1.16 (2.58)<br>[0, 0, 7.95]
	ln(Buildings)	Natural logarithm of one plus the total square meters of building footprints within one kilometer as of 2010	Authors’ calculations using Chicago Metropolitan Agency for Planning (2014)	5.39 (2.52)<br>[0, 6.46, 7.73]
	ln(Roads)	Natural logarithm of one plus the total square meters of roads or railroads within one kilometer as of 2010	Authors’ calculations using Chicago Metropolitan Agency for Planning (2014)	5.5 (2.65)<br>[0, 6.51, 7.92]
	ln(Paved)	Natural logarithm of one plus the total square meters of other paved areas within one kilometer as of 2010	Authors’ calculations using Chicago Metropolitan Agency for Planning (2014)	5.92 (2.02)<br>[0, 6.64, 8.23]

Table S2. Best-fitting logistic regression models for diurnal and nocturnal species occurrence at camera traps. * = sig. at 0.05; ** = sig. at 0.01; *** = sig. at 0.001.

Variable	Diurnal Species Occurrence	Nocturnal Species Occurrence
Intercept	-5.79***<br>(1.11)	-8.54***<br>(1.78)
High Illumination	0.979***<br>(0.181)	0.777*<br>(0.389)
Time Since Dusk	4.75***<br>(0.370)	-2.70***<br>(0.409)
Time Since Dusk^2	-4.64***<br>(0.355)	1.92***<br>(0.250)
Time Since Dusk^3	1.83***<br>(0.146)	-0.406***<br>(0.0554)
Time Since Dusk^4	-0.313***<br>(0.0265)	0.0270***<br>(0.00409)
Time Since Dusk^5	0.0190***<br>(0.00174)	
Average Humans	0.283***<br>(0.0134)	-0.549***<br>(0.0504)
ln(Distance from Loop)	-0.158***<br>(0.0268)	0.371***<br>(0.0547)
Length of Day	1.49***<br>(0.146)	0.911***<br>(0.227)
Length of Day^2	-0.0680***<br>(0.00593)	-0.0395***<br>(0.00924)
Moonlight	-0.212<br>(0.170)	0.550*<br>(0.240)
Daylight	1.32***<br>(0.0457)	-1.42***<br>(0.0881)
ln(Light Residential)	-0.373***<br>(0.0338)	0.151***<br>(0.00882)
ln(Dense Residential)	-0.885***<br>(0.0332)	-0.0908<br>(0.0592)

ln(Commercial)	-0.307***<br>(0.0207)	0.416***<br>(0.0334)
ln(Industrial)	-0.0404**<br>(0.0149)	-0.116***<br>(0.0155)
ln(Tree Cover)	0.849***<br>(0.0258)	-0.0961**<br>(0.0369)
ln(Grass)	0.151***<br>(0.0188)	-0.396***<br>(0.0224)
ln(Soil)	0.0227***<br>(0.00497)	-0.0751***<br>(0.00845)
ln(Water)	0.0823***<br>(0.00706)	-0.0669***<br>(0.0111)
ln(Buildings)	0.311***<br>(0.0105)	-0.0498***<br>(0.0117)
ln(Roads)	-0.0174*<br>(0.00775)	-0.241***<br>(0.0128)
ln(Paved)	-0.0356**<br>(0.0132)	0.172***<br>(0.0213)
High Illumination*Time Since Dusk	-0.958<br>(0.518)	0.586<br>(0.821)
High Illumination*Time Since Dusk^2	0.878<br>(0.503)	-0.523<br>(0.489)
High Illumination*Time Since Dusk^3	-0.385<br>(0.208)	0.126<br>(0.108)
High Illumination*Time Since Dusk^4	0.0789*<br>(0.0378)	-0.00909<br>(0.00796)
High Illumination*Time Since Dusk^5	-0.00584*<br>(0.00250)	
McFadden’s Pseudo R^2	0.141	0.166
Area under the ROC Curve	0.793	0.842
N (Cameras)	62	62
N (Hours)	240,696	240,696

Table S3. Descriptions of landscape metrics calculated for greenspace in Chicago.

**Landscape metrics**	**Description**
Total Land Area	Sum of areas of all patches in the landscape
Number of Patches	Total number of patches
Mean Patch Size	Average patch size
Patch Size Standard Deviation	Standard Deviation of patch areas.
Mean Nearest Neighbor	Measure of patch isolation. The nearest neighbor distance of an individual patch is the shortest distance to a similar patch (edge to edge).
Edge Density	Amount of edge relative to the landscape area
Total Edge	Perimeter of patches
