# Supplementary material for: Mapping behaviorally relevant light pollution levels to improve urban habitat planning
Source: Sci Rep. 2019 Aug 15;9:11925. doi: 10.1038/s41598-019-48118-z (PMC6695421; doi:10.1038/s41598-019-48118-z)
Supplement: Supplementary file 2 — Data and Replication Package [file 41598_2019_48118_MOESM2_ESM.zip › Replication Materials/Replication Notes README.pdf]

Mapping behaviorally relevant light pollution levels to improve urban habitat planning replication notes

Laboratory Analysis

The R code file “LabbehavioranalysisRpack.R” in the replication folder contains a script for reproducing statistical analyses of output from the behavioral experiments conducted in the lab, data for which is contained in the “LaboratoryData.csv” file. In-line annotations in the R-code explain the operations conducted. Column headers in the “LaboratoryData.csv” file correspond to the following variables:

Header	Variable
Light level	Grouping variable designating the illumination levels, in lux, tested in the lab.
ID	Unique identifier for each subject.
Tau1_(ChiSq)	Period measurement, as a Chi-squared value.
Err_Onset	Onset error in hours.
Total_Counts	Total wheel rotations during the period.
Alpha	Alpha (activity) hours.

Illumination Estimation with Regression Trees

The R code file “RegressionTreeModels.R” contains a script to replicate the regression-tree models used to estimate ground-level illumination across the city. The first several lines (up to 203) create functions to aggregate pixel values to buffered points than can be done using R’s raster package. The remaining code is explained using in-line comments. This script calls several datasets included in the replication package. The first, “ControlPts.csv” records the location and illumination measurements, in lux, conducted at groundtruthing points used to fit the regression tree models. This file contains three columns, as follow:

Header	Variable
lat	Latitude coordinate, in WGS84.
lon	Longitude coordinate, in WGS84.

lux	Measured illumination level, in lux.

In line 239, the script loads raster images taken from georeferenced astronaut photography from the International Space Station. These images are found in “2008g_modified.tif” and “chclr2013.tif”.

On line 389, the code reads in a set of gridded (“RegPointswnCity.shp”) and randomly generated points (“PredictPoints.shp”), both in ESRI Shapefile format, which are then joined and used as the prediction points for the regression tree models.

The rest of the code prepares the data and then estimates the regression tree models, saving these to the working directory as a shapefile, “PredictedLuxPointsCity.shp”).

Camera Trap Comparisons

Because the camera traps from which our data are drawn are still in use, we cannot disclose the precise locations without risking ongoing observational research. Instead, we provide all the data we collected or computed using camera locations, along with code to replicate our results. Code for replicating Figure 3A is found in the “KernalDensityAnalysis_Diurnal.R” and “KernalDensityAnalysis_Nocturnal.R” files, for diurnal and nocturnal species, respectively. Both scripts read in raw data from the camera traps, found in the "photo_pollution_with_rad_extended_11_13.txt" file. This file contains the following columns:

Header	Variable
SurveyID	Unique identifier for the camera trap and season deployed.
Date	Day the image was taken.
Time	Time the image was taken.
Species	Human-coded species observed in the image.
N_ind	Number of individuals of the species observed in the image.
StationID	Unique identifier for each camera trap.
Season	Survey deployment season.
Year	Year of deployment.
Time_rad	Time of day image was taken, in radians.

Operations used to conduct this analysis are explained in in-line comments in the script.

Logistic Regression Models

The “Field Data Replication.R” file contains a script used to prepare data and estimate the logistic regression models used to generate Figure 3B. The script reads in three data sources, “TrapPhotos.csv”, “TrapGeographicData.csv”, and “TrapPredictions.csv”. These include the following columns:

TrapPhotos.csv

Column	Variable
Species	Human-coded species observed in the image.
TimeBin	Hour of the day in which the image was taken, used to group observations.
SurveyID	Unique identifier for the camera trap and season deployed.
Date	Day the image was taken.
N_ind	Number of individuals of the species observed in the image.
Season	Survey deployment season.
Year	Year of deployment.
Time_rad	Time of day image was taken, in radians.
Degree	Time of day image was taken, in degrees.
CT.1	Time image was taken.
CT.2	Time image was taken.
SiteID	Unique ID for general camera trap location.
SiteName	Place name of general camera trap location.
Distance	Distance of buffer, in meters.
Sum	Sum of illumination, in lux, within buffer.
Mean	Mean of illumination, in lux, within buffer.
Median	Median of illumination, in lux, within buffer.

Maximum	Maximum of illumination, in lux, within buffer.
Level	Light level assigned to camera trap in kernal density analysis.
TimeofDay	Qualitative time of day image was taken.

TrapGeographicData.csv

Column	Variable
SiteName	Place name of general camera trap location.
SiteID	Unique ID for general camera trap location.
Transect	Trap location indicator.
Section	Trap location indicator.
StationID	Unique identifier for each camera trap.
Dist	Distance from Chicago central business district, in kilometers.
LCCode1	Tree cover within one kilometer, in square meters.
LCCode2	Grass cover within one kilometer, in square meters.
LCCode3	Bare soil cover within one kilometer, in square meters.
LCCode4	Water land cover within one kilometer, in square meters.
LCCode5	Built-up land cover within one kilometer, in square meters.
LCCode6	Road land cover within one kilometer, in square meters.
LCCode7	Paved, non-road land cover within one kilometer, in square meters.
DenseRes	Dense residentially zoned areas within one kilometer, in square meters.
LightRes	Light residentially zoned areas within one kilometer, in square meters.
Commercial	Commercially zoned areas within one kilometer, in square meters.
Industrial	Industrially zoned areas within one kilometer, in square meters.

TrapPredictions.csv

Column	Variable
SiteID	Unique ID for general camera trap location.
StationID	Unique identifier for each camera trap.
SiteName	Place name of general camera trap location.
Distance	Distance of buffer, in meters.
Sum	Sum of illumination, in lux, within buffer.
Mean	Mean of illumination, in lux, within buffer.
Median	Median of illumination, in lux, within buffer.
Maximum	Maximum of illumination, in lux, within buffer.
Level	Light level assigned to camera trap in kernal density analysis.
TimeofDay	Qualitative time of day image was taken.

Until line 558, the code prepares the data for model estimation, find the optimal logistic regression model, and generates the figures summarizing model output used in the article. Each step is explained using in-line comments in the code.

Lines 561 through 593 create Figure 5. Because the summary variables are few in number and were calculated using output from FragStats, they are written into the code itself.

Lines 600 through the end of the script generate FigureS5. They read in one last dataset, "NeighborhoodPercents.csv", which includes the percentage of greenspace in each official Chicago neighborhood illuminated at greater than 6 lux. This file contains the following columns:

Column	Variable
PRI_NEIGH	Official neighborhood name
percent	Percentage of greenspace in the neighborhood estimated to be illuminated at greater than 6 lux.
