# Supplementary figures and images for: Mapping behaviorally relevant light pollution levels to improve urban habitat planning
Source: Sci Rep. 2019 Aug 15;9:11925. doi: 10.1038/s41598-019-48118-z (PMC6695421; doi:10.1038/s41598-019-48118-z)

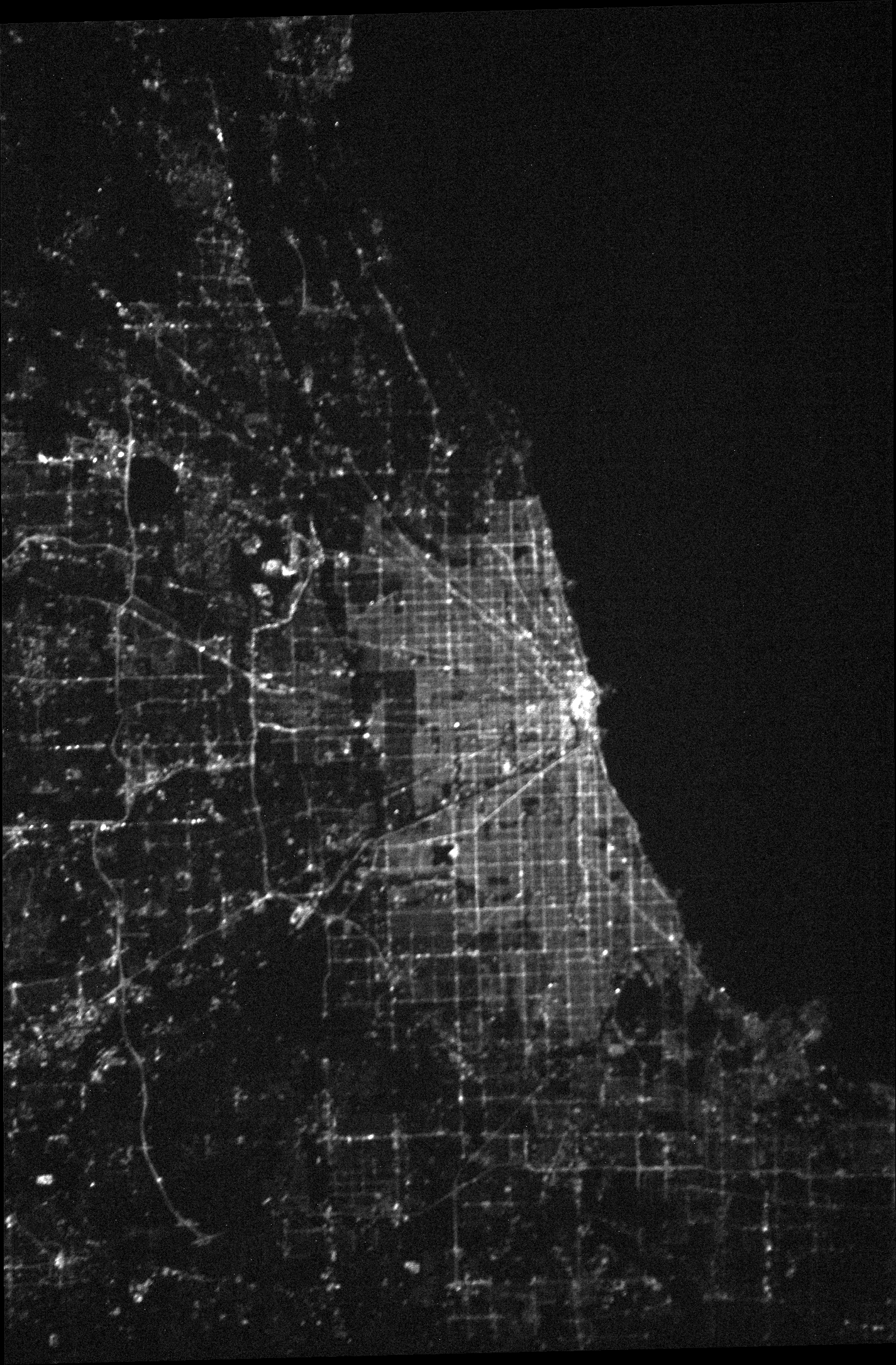

Supplement: Supplementary file 2 — Data and Replication Package [file 41598_2019_48118_MOESM2_ESM.zip › Replication Materials/2008g_modified.tif]

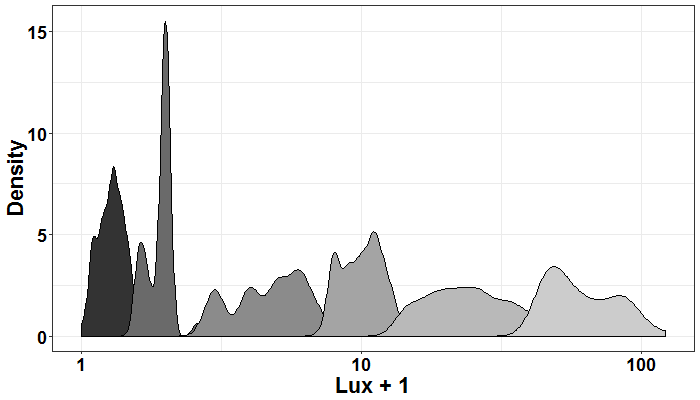

Supplement: Supplementary file 2 — Data and Replication Package [file 41598_2019_48118_MOESM2_ESM.zip › Replication Materials/ClusterDensityNS.png]
